# Endothelial Dysfunction and Chronic Inflammation: The Cornerstones of Vascular Alterations in Age-Related Diseases

**DOI:** 10.3390/ijms232415722

**Published:** 2022-12-11

**Authors:** Gaetano Pacinella, Anna Maria Ciaccio, Antonino Tuttolomondo

**Affiliations:** Internal Medicine and Stroke Care Ward, Department of Promoting Health, Maternal-Infant, Excellence and Internal and Specialized Medicine (PROMISE) G. D’Alessandro, University of Palermo, Piazza delle Cliniche n.2, 90127 Palermo, Italy

**Keywords:** endothelial dysfunction, cellular senescence, inflamm-ageing, vascular alterations, chronic inflammation

## Abstract

Vascular diseases of the elderly are a topic of enormous interest in clinical practice, as they have great epidemiological significance and lead to ever-increasing healthcare expenditures. The mechanisms underlying these pathologies have been increasingly characterized over the years. It has emerged that endothelial dysfunction and chronic inflammation play a diriment role among the most relevant pathophysiological mechanisms. As one can easily imagine, various processes occur during aging, and several pathways undergo irreversible alterations that can promote the decline and aberrations that trigger the diseases above. Endothelial dysfunction and aging of circulating and resident cells are the main characteristics of the aged organism; they represent the framework within which an enormous array of molecular abnormalities occur and contribute to accelerating and perpetuating the decline of organs and tissues. Recognizing and detailing each of these dysfunctional pathways is helpful for therapeutic purposes, as it allows one to hypothesize the possibility of tailoring interventions to the damaged mechanism and hypothetically limiting the cascade of events that drive the onset of these diseases. With this paper, we have reviewed the scientific literature, analysing the pathophysiological basis of the vascular diseases of the elderly and pausing to reflect on attempts to interrupt the vicious cycle that connotes the diseases of aging, laying the groundwork for therapeutic reasoning and expanding the field of scientific research by moving from a solid foundation.

## 1. Introduction

The pathologies with the most significant epidemiological impact nowadays, not least because of longer life spans and a rise in the average age, are vascular diseases that affect elderly individuals. Therefore, the characterization of the physiopathological mechanisms underlying them is of primary importance, intending to slow down and, where possible, counteract the onset of these illnesses, which is why scientific research, in recent decades, has increasingly attempted to recognize the predisposing and precipitating factors of age-related pathologies: among these, endothelial dysfunction and chronic age-related inflammation certainly stand out.

Our work reviews scientific literature and delves into the most exciting aspects of vascular diseases in the elderly. We focused, mainly, on those mechanisms that are the object of study for the development of effective therapies. These represent the only weapon to counter the inexorable decline of the human body, trying to increase the quality of life as well as the quantity, and preventing many of those adverse outcomes linked to cardio and cerebrovascular accidents.

Before discussing its dysfunction and the consequent alterations, it is necessary to digress into the structural and functional characteristics of the endothelium, formerly known as the “cellophane wrapper”, of the vascular system, consisting of a monolayer of specialized endothelial cells which line the luminal surface of the whole circulatory system [1].

Beyond its well-known role as a physical barrier between blood and tissues, it is regarded as a dynamic and multifunctional endocrine organ [2]. It plays pivotal functions in maintaining homeostasis, such as blood filtration, blood vessel tone modulation, hemostasis, immune response regulation, angiogenesis, and hormone trafficking [3].

Specifically, the endothelium is a crucial player in orchestrating vascular function; consequently, endothelial dysfunction represents the first step in vascular alteration. The latter is characterized by well-defined phenotypic alterations, including a vasoconstrictive, prothrombotic, and proinflammatory state, as well as changes in the extracellular matrix influencing vascular stability [4,5,6].

The impaired endothelial vasodilatation is mainly the result of the reduced activity of endothelial oxide nitric synthase (eNOS), leading to reduce levels of oxide nitric (NO), which represents the most important regulator of vasodilatation. It also exerts vasoprotective and cardioprotective effects. Robust evidence on the fundamental role of NO in cardiovascular protection is derived from experimental studies on eNOS knock-out mice that develop premature aging phenotypes, leading to early mortality [6].

The progressive decline of endothelial cells is a hallmark of physiological ageing. Several animal and human experimental studies showed that inflammation is one of the essential pathological processes underpinning endothelial dysfunction [7]. In addition, a chronic low-grade inflammation, termed “inflamm-ageing”, is, in fact, another feature of ageing [1,8]. Inflamm-ageing is defined as a sterile and non-resolving inflammation that progressively increases with biological aging. It was initially thought to result from “continuous antigenic load and stress”, but in the last decades, much evidence has contributed to depicting a complex phenomenon involving several mechanisms which we will characterize in this work.

Certain mechanisms involved in the physiological aging of the organism, and thus in vascular aging, such as telomere shortening, loss of proteostasis, accumulation of mutations to mitochondrial DNA, and alteration of nuclear DNA damage repair mechanisms, are also responsible, under specific circumstances, for the onset of pathologies characterized by premature aging like progeroid syndromes, in which the pathophysiological trigger is represented by an alteration that results in a premature defect in the functioning of the mechanisms mentioned above. All this suggests that aging is promoted by a modification of molecular processes at different levels, the accumulation of such alterations promotes its rapid progression, and the resulting inflammation deteriorates organs and tissues the more rapidly the more it is represented [9].

Confirming the above, studies in animal models have revealed that vascular aging is earlier the more some of the mechanisms that regulate the response to vasomodulating substances are altered: in knockout animals for some genes involved in nuclear DNA damage repair mechanisms of vascular smooth muscle cells, nonatherosclerotic decay was seen due to altered endothelial responses [10,11].

Endothelial dysfunction and inflamm-ageing, as mentioned above, represent two critical mutually linked mechanisms underlying vascular alterations leading to age-related diseases. In the 17th century, the physician Thomas Sydenham, called the “English Hippocrates”, stated that “*a man is as old as his arteries*” [12]. Indeed, vascular alterations play a crucial causal role in cardio- and cerebrovascular diseases, including stroke, atherosclerosis, and coronary artery disease, representing the leading causes of disability and mortality among older individuals. Additionally, vascular alterations significantly contribute to the development of common age-related disorders, including erectile dysfunction, renal dysfunction, Alzheimer’s disease, circadian cycle alterations, Myocardial Infarction With Nonobstructive Coronary Arteries (MINOCA), HFpEF, and osteoporosis [5].

This review summarizes experimental and clinical evidence on the complex interplay among ageing, endothelial dysfunction, inflamm-ageing, and vascular diseases.

## 2. Cellular Senescence and Inflamm-Ageing

The “inflamm-ageing”, as stated above, is a recently coined term referring to a sterile, non-resolving, low-grade, and chronic inflammation that progressively increases with age [8,13]. It is characterized by chronic activation of the innate immune system associated with persistently increased levels of proinflammatory mediators, such as C-reactive protein (CRP), mannose-binding lectin (MBP), and cytokines that have a more inflammatory role such as interleukin IL-1 β, IL-6, and tumor necrosis factor (TNF)-α [14].

Inflammation, under normal conditions, is a fundamental defence mechanism by which the immune system recognizes and removes harmful and foreign stimuli, such as traumatic tissue injury or an invading pathogen [15]. It can be classified as acute, which represents a physiological mechanism for restoring homeostasis, or chronic, which is persistent, resulting in responses that lead to tissue degeneration. During ageing, several factors can contribute to the activation of chronic inflammation. The underlying causes are still unclear, but several cellular and molecular mechanisms, which are synergistic and mutually promoting, have been postulated. Franceschi et al. suggested that the development and maintenance of the inflamm-ageing is not the result of a punctual phenomenon but of the individual immunobiography, defined as the immune history of everyone [16].

During life, individuals are exposed to many stressors, such as pathogens, nutrients, vaccines, and cellular debris, evoking an immune response with different characteristics, especially in intensity and duration. A feature of the immune system is plasticity, which makes immune cells able to modulate the response according to the type, the dose, and the temporal sequence of the stimuli. The immunobiography of each individual is unique and results from the summation and interaction of all the immunological experiences [16]. The immunobiography could represent the basis of the age-related changes in the immune system and, consequently, which makes the inflamm-ageing comprehensible and plausible. In advanced age, the immune system undergoes remodeling, characterized on one side by loss of functions and activities predisposing to an increased risk of infections and autoimmune diseases, and on the other side by the chronic activation of the innate response leading to the constant production of inflammatory cytokines. The increased levels of cytokines were first described in an in vitro study on stimulated peripheral blood mononuclear cells from elderly donors [17]. Then, it was confirmed by several literature pieces of evidence [18,19,20]. Beyond immunobiography, inflamm-ageing is promoted by different molecular age-related mechanisms, including activation of the DNA damage response (DDR), cellular senescence, mitochondrial dysfunction, defective autophagy, and mitophagy. The latter represents a driving force with a crucial role [21].

Regarding mitochondrial damage, increasingly clear evidence suggests that it plays a role in promoting age-related inflammation. It is now widely known that mitochondria play pleiotropic functions beyond energy production, including cell signaling pathways, cell survival, and apoptosis. Mitochondrial functions are fundamental for all human organs and tissues to maintain homeostasis and oxygenation [22]. Mitochondrial DNA (mtDNA) is more prone to damage than genomic DNA, which is protected and stabilized by histones. The mtDNA is particularly susceptible to oxidative damage, located close to the electron transport chain.

Additionally, mtDNA is present in many copies and is replicated more frequently than genomic DNA, leading to a more frequent rate of replication errors; in addition, mtDNA polymerase gamma (Polγ) is associated with a higher error rate. All these mechanisms underpin the increased rate of mtDNA mutagenesis and accumulation of mtDNA mutations, which lead to mitochondrial dysfunction: ageing is characterized by a progressive increase in mitochondrial dysfunction. Interestingly, an intimate relationship between mitochondrial dysfunction and inflammation exists. Indeed, altered mitochondria release molecules acting as damage-associated molecular patterns (DAMPs) and thus are recognized by PRR on innate immune system cells. Among mitochondrial DAMPs, mtDNA is involved in NLRP3 inflammasome activation [23].

### 2.1. Autophagy

Another mechanism with a prominent role in inflamm-ageing is defective autophagy. It represents a crucial lysosomal process for taking down cellular contents, preserving energy, and preventing the accumulation of damaged and aggregated molecules. It is possible to distinguish three different pathways: micro-autophagy, chaperone-mediated autophagy, and macroautophagy. Among these, macro-autophagy is the most well-characterized: it occurs by forming membrane vesicles termed “autophagosomes”, followed by the engulfment of cytoplasmic cargo within autophagosomes and the fusion of autophagosome and lysosome, leading to the formation of “autophagolysosome”, which degrades macromolecular substrates in smaller molecules. Thus, these mechanisms contribute to cellular homeostasis and metabolism.

The chaperone-mediated autophagy (CMA) is specifically involved in the degradation of a substrate with “KFERQ amino acid recognition sequence”, the targeting motif in the protein. The first step in CMA is the binding between the target protein and Hsc70. Next, this complex interacts with CMA receptor LAMP-2A, which will be internalized in two lysosomes and finally degraded.

Micro-autophagy was first described in 1966 in yeast and was detected in mammals only in 2011; there is still limited evidence of its biological functions and underlying molecular mechanisms. Micro-autophagy relies on the entrapment and transport of cytosolic cargo into the lumen of the lytic organelles. Specifically, it has been proposed that three types of micro-autophagy exist: type 1, characterized by the lysosomal protrusion, type 2, characterized by lysosomal invagination, and type 3, characterized by endosomal invagination.

### 2.2. Mitophagy

Mitophagy, the selecting autophagy of the mitochondria, is another crucial mechanism for checking the mitochondrial quality by eliminating altered mitochondria. Additionally, it contributes to the elimination of mitochondria from maturing erythrocytes and sperm-derived mitochondria following fertilization. Impaired autophagy and mitophagy are involved in the pathogenesis of several ageing-related diseases. Both autophagy and mitophagy activity decline with age and noteworthy mitochondria undergo structural and functional alterations, which cannot be efficiently removed. In addition, a link between inflammation and autophagy has recently emerged. Several studies showed that autophagy could suppress inflammatory reactions. Mitochondria with disrupted integrity and impaired autophagic clearance are the crucial regulators of inflammasomal activation and inflammatory responses.

Autophagy and mitophagy are fundamental mechanisms for maintaining tissue homeostasis. However, the efficacy of these mechanisms progressively declines during ageing, leading to the accumulation of dysfunctional mitochondria and cellular debris. Furthermore, it has been shown that defects in mitochondrial degradation could induce ROS production and stimulate inflammasomes.

Experimental trials in mice showed that the genetic inhibition of autophagy stimulates age-related alterations and reduces the lifetime. Additionally, some authors documented that improving the autophagic capacity by pharmacological stimuli may prevent age-related disease and extend lifespan [24].

### 2.3. Cellular Senescence

Cellular senescence was first described in 1965 as a process to limit the proliferation of human cells in vitro [25]. It represents the complex set of mechanisms by which each cell undergoes irreversible changes that result in its aging and the tissue of which it is a part.

#### 2.3.1. Causative Mechanisms

Several decades after, the mechanisms underlying cellular senescence have been elucidated: it is defined as the irreversible cell cycle arrest in response to a stimulus, which leads to phenotypical and functional alterations involving cell morphology, metabolism, chromatin organization, gene expression, and activation of a proinflammatory secretome, termed as SASP (senescence-associated secretory phenotype), which includes cytokines, chemokines, growth factors, and tissue-remodeling enzymes [26].

Under physiological situations, cellular senescence has a critical role in regulating cell proliferation, limiting malignant transformation, promoting tissue remodeling during embryonic development, and wound healing. The clearance of senescent cells occurs by apoptosis or is mediated by the immune system. However, the ageing of the immune system reduces the recognition capacity of senescent cells, which, together with SAPS-dependent mechanisms, leads to the accumulation of senescent cells. Additionally, the proinflammatory and oxidative environment typical of ageing due to the production of reactive oxygen species as a result of an inflammatory setting stimulates the nuclear factor-kappa-light-chain enhancer of activated B cells (NF-κB) pathway, which represents an essential regulator of inflammation [27].

#### 2.3.2. Mechanisms of Cell Cycle Arrest

From a biochemical point of view, senescence can occur through two different mechanisms: telomere-dependent replicative senescence, also known as “Hayflick’s limit”, or stress-induced premature senescence (SIPS) [28]. The first occurs when a cell’s division capacity has been exhausted, leading to the shortening of telomeres and, consequently, to an irreversible state of proliferation arrest. Such a mechanism is fundamental for tumor suppression [29].

Several mechanisms can stimulate telomeric DNA loss; among these, oxidative stress is a significant contributor. The SIPS, instead, is a telomere shortening-independent mechanism and can be induced by different stimuli, such as inflammation and oxidative stress: it is characterized by DNA damage followed by the activation of the DNA damage response (DDR), a set of different mechanisms (including DNA repair processes, damage tolerance processes, cell cycle checkpoints to ensure genome integrity, transcription stalling—due to gridlock of RNA polymerase caused by DNA damage—and metabolic reprogramming—a change in metabolic patterning that promotes the onset of molecular alterations) [30,31] that plays a decisive role in determining the fate of a cell, directing it toward DNA repair or apoptosis [23]. Erroneously repaired DNA lesions could result in mutations, while unrepaired damage could induce cellular senescence or apoptosis. Dysregulation of DDR is associated with cancer susceptibility, accelerated ageing, and developmental abnormalities [32].

#### 2.3.3. Relation to Nutrient Sensing/NADH

The factors that drive the processes of cellular senescence are multiple, as one can easily guess, and defects acquired in the context of different metabolic pathways each play a significant role. It can be precipitated by the alteration of molecular processes of specific pathways involved in the regulation of cellular homeostasis, including the NAD^+^/NADH pathway (involved in countless functions including energy regulation of cells and balance of oxidative stress), the mitochondrial dysfunction, the mechanistic target of the rapamycin (mTOR) network, and the “silencing information regulator two related enzyme 1” (sirtuin1, SIRT1) signaling [28]. Cell growth arrest is mediated by the activation of cyclin-dependent kinase inhibitors that block the phosphorylation of retinoblastoma-associated protein (RP) and induce cell cycle blockade [33].

The RP phosphorylation occurs through the p21/p53 or the p16^INK4A^ pathways.

#### 2.3.4. Relation to SAPS/Inflammation

The SASP secretome produced by senescent cells (SCs) is cell-type specific. It can act both in a paracrine manner, triggering the senescence in neighbouring cells, and in an endocrine manner, contributing to inflamm-ageing.

Several studies revealed that ageing is associated with the progressive accumulation of senescent cells in different organs and tissues, including skin, atherosclerotic plaque, and endothelium. They contribute to age-related disease onset [34,35,36,37].

In vitro trials have shown that, in addition to the mechanisms already described, several conditions may trigger cellular senescence: hypoxia, oxidative stress, beta-amyloid peptide precipitation, and chronic inflammatory disorders [38,39,40].

Senescent cells that have gone through the aging processes mentioned earlier have been proposed to contribute significantly to inflamm-ageing by different mechanisms [8,13,41]. First, the production of SASP has a prominent role for the reasons described above [13]. Additionally, cellular debris released by SCs is involved in promoting inflamm-ageing: they include a wide range of damage-associated molecular patterns (DAMPs) that stimulate cell surface and intracellular “pattern recognition receptors” (PRRs), which transduce signals to trigger a proinflammatory response, primarily in macrophages and mast cells [13]. Specifically, DAMPs include high-mobility group B1 (HMGB1) protein, sodium monourate and uric acid crystals, and oxidized fatty acids, such as oxidized LDL, and other proteins. In addition, the activated innate immune system cells stimulate the classical proinflammatory pathways, leading to the production and secretion of cytokines, such as IL-6.

The ageing also leads to the senescence of the tissue-resident stem cells, including mesenchymal stromal cells (MSCs). Aged MSC could contribute to the development of inflamm-ageing through the production of cellular waste, the acquisition of SASP, the alteration of their immunomodulating capacity, and the impairment of their function in maintaining hematopoietic stem cells’ homeostasis and bone marrow microenvironment [13].

#### 2.3.5. Cellular Senescence and Inflammasome

Cellular aging is, therefore, the result of all those mechanisms listed so far, of that vicious cycle between chronic inflammation, oxidative stress, and permanent cellular damage that feed off each other. In this setting, inflammasome has a prominent role [23]. It is an intracellular macromolecular complex that, upon activation induced by different endogenous and exogenous stimuli, orchestrates an inflammatory response, leading to proinflammatory cytokines. Among inflammasomes, NOD-like receptors protein 3 (NLRP3) is the most investigated and characterized [41]. NLRP3 inflammasome consists of NLRP3, ASC (the adaptor molecule apoptosis-associated speck-like protein containing a CARD), and pro-caspase 1. The extrication of NLRP3 occurs in two steps: priming and activation. The priming leads to the NF-κB-mediated NLRP3 and pro-IL-1 β expression. At the same time, the activation step, triggered by pathogen-activated molecular patterns (PAMPs) and DAMPs, promotes NLRP3 inflammasome installation and caspase-1-mediated IL-1 β and IL-18 release and pyroptosis [23]. The importance of NLRP3 inflammasome in inflamm-ageing is supported by the evidence that its inhibition is associated with an enhanced health span and reduced age-dependent degenerative changes; its activity and enhancement, instead, is related to an increased risk of age-related disorders, such as atherosclerosis and neurodegenerative diseases [42].

In conclusion, inflamma-ageing predisposes the development of chronic, non-transmissible diseases such as diabetes, cancer, and cardiovascular diseases [14].

Inflamm-ageing has emerged as an essential risk factor for morbidity and mortality in the elderly.

## 3. Endothelial Dysfunction and Impaired Angiogenesis in the Elderly

Age-related endothelial dysfunction is associated with the structural impairment of the circulatory system. Microvascular homeostasis results from the dynamic balance between pro-angiogenic and anti-angiogenic factors. Accumulating evidence suggests that aging is characterized by altered angiogenic processes leading to a progressive deterioration of microvascular homeostasis and thus is predisposed to the development of cardiovascular diseases, such as hypertension, diabetes, atherosclerosis, and cerebrovascular diseases.

Angiogenesis, defined as forming new vessels from pre-existing functional vessels, is necessary for physiological and pathological conditions. It is essential during embryogenesis, cardiovascular maturation, and tissue development and repair [43]. It also involves many pathological processes, such as tumor progression and chronic inflammation. Angiogenesis is a dynamic, complex mechanism involving several actors, such as endothelial cells, pericytes, and growth factors; it occurs in tiny capillaries and implicates the sprouting of existing endothelial cells. Specifically, the driving mechanism is the arrangement of endothelial cells in tip and stalk cells. Tip cells make up filopodia, which invade the surrounding tissue guiding the formation of new vessels [44]. This process is regulated by the interplay between the Vascular Endothelial Growth Factor (VEGF) and Notch signaling [45]. Tip cells abundantly express receptors for VEGF (VEGFR), and the activation of the VEGF pathway leads to filopodia formation, conferring the sprouting phenotype to tip cells. However, the VEGF pathway also induces the activation of the Notch signaling in the neighbouring stalk cells, which inhibits the VEGFR expression, conferring them the non-sprouting phenotype [46]. Thus, tip cells are characterized by high VEGF and low Notch expression, while the stalk cells have low VEGF and high Notch expression. Notch also has a pivotal role in lumen morphogenesis and the acquisition of barrier characteristics. Under physiological conditions, the balance between VEGF and Notch signaling is strictly regulated, forming organized structures. Beyond VEGF and Notch pathways, several factors, directly or indirectly, control endothelial proliferation, sprouting, and maturation. Among these, the most important are Ang-Tie, BMP/TGF-β, EphrinB2-EphB4, Cxcr4-Cxcl12, and Wnt [47].

Pericytes also represent important players in angiogenesis by modulating endothelial cell proliferation and migration [48]. Additionally, they have a crucial role in regulating endothelial dynamics and capillary function.

Noteworthy, the angiogenic function is progressively lost during aging, as evidenced in animal trials and clinical studies. For example, experimental models showed that old animals have a reduced capability for collateral vessel development in response to ischemia [49]. Similarly, older patients with coronary artery disease (especially arterial obstructive vascular disease) and consequent congestive heart failure have a reduced capacity to form collateral arteries [50].

Additionally, in vitro studies showed that endothelial cells from old mice have reduced proliferating and migrating capacity [51]. Another exciting piece of evidence is that, although cancer incidence increases with age, the progression is slowed down in older individuals compared to younger individuals. This could be partly explained by the impairment of angiogenesis, which represents the driving process for tumor growth and metastasis, as confirmed in experimental studies.

Several mechanisms have been hypothesized to underpin the age-related impairment of angiogenesis. First, the senescence of endothelial cells is associated with significant changes in gene expression, cellular replication, and morphological phenotypes, compromising the endothelium integrity and, consequently, the angiogenesis. The molecular mechanisms underlying endothelial cell senescence have yet to be fully understood. However, cell cycle deregulation, oxidative stress, impaired calcium signaling, and inflammation could be involved. Robust evidence highlights a prominent role, specifically in oxidative stress. It is defined as the consequence of the imbalance between the production and removal of reactive oxygen and nitrogen species (ROS and RNS, respectively) [52]. Much evidence has confirmed that free radicals are dangerous, and many mechanisms have been proposed to elucidate the relationship between oxidative stress and aging [53]. ROS and RNS include unstable free radicals, such as hydroxyl (OH^−^), superoxide anions (O_2_^−^), nitric oxide (NO^−^) radicals, and non-free radicals, such as hydrogen peroxide (H_2_O_2_). They are highly reactive, unstable species and can modify several substrates, including DNA, proteins, and lipids. The primary source of ROS is mitochondria, but peroxisomes, microsomes, and immune system cells also contribute to their production. Under physiological conditions, ROS plays an essential biological function within different cellular processes. However, the increase in their concentrations has several adverse effects when inducing impaired cell structure and functions, resulting in senescence [54]. The increase in ROS is generally neutralized by antioxidants, which can be classified as enzymatic, such as superoxide dismutase (SOD), glutathione peroxidase (GPX), catalase (CAT), and thioredoxin (Trx), and non-enzymatic, such as carotenoids, ubiquinol, and flavonoids. The imbalance between ROS and antioxidants leads to oxidative stress.

Another important mechanism involved in age-related impaired angiogenesis is the alteration of growth factors. Experimental studies showed that old mice had reduced levels of VEGF and capillary-to-fiber perimeter exchange than young mice [55]. The restoration of VEGF signaling in aged animals by transfecting the VEGF121 gene could rescue the age-dependent decline in angiogenesis [56]. Overall, cumulating literature evidence shows that aging is characterized by the reduction of VEGF levels, which could be due to the impaired activation of the transcription factor for VEGF, namely hypoxia-inducible factor-1α [55].

Interestingly, pericytes could also contribute to the impairment of angiogenesis. During aging, pericytes undergo ultrastructural changes that could compromise their function [57]. Some authors also described a reduction in pericytes in aged monkeys and rats. In contrast, others reported increased pericytes or no changes [57,58]. Thus, findings on the possible role of pericytes in age-related endothelial and angiogenic dysfunction are still controversial, and further studies are required to elucidate them.

The decrease of nitric oxide (NO) is another hallmark of aging, with a pivotal role in endothelial dysfunction and angiogenic alteration. NO is a crucial regulator of endothelial homeostasis and has multiple biological functions, including stimulating VEGF secretion from endothelial cells and macrophages [59]. Chin et al., in in vivo murine models, showed that the inhibition of endothelial nitric oxide synthase (eNOS) was associated with the impairment of angiogenesis not because of aging per se but because of all the mechanisms (including endothelial dysfunction, smooth muscle cell disruption, increased oxidative stress, and DNA damage) that mark it [60,61]. Although eNOS represents the primary source of NO in the vascular endothelium, neuronal NOS (nNOS) also contributes to endothelial cells-derived NO. Indeed, the constitutive expression of nNOS has also been described in vascular endothelial cells [62,63]. Similarly to eNOS, nNOS decreases during aging, contributing to the age-related reduction of NO.

In summary, several mechanisms have been proposed to explain the relationship among endothelial dysfunction, impaired angiogenesis, and aging, but there is a long way to go. The discovery of mechanisms underpinnings age-related angiogenesis impairment is hampered by the difficulty in obtaining reliable in vivo and in vitro that can reflect the authentic setting of the elderly [64]. Indeed, there is no gold standard for angiogenic models in the field of aging research. Many questions remain to be answered, as brilliantly described in the review by Hodges et al.

### 3.1. Endothelial Dysfunction in Hypertension

Arterial hypertension, defined as a persistent increase in arterial pressure, represents a leading modifiable risk factor for cardiovascular diseases and premature death worldwide. Furthermore, the global burden of hypertension is growing due to population aging, which is expected to affect one-third of the population worldwide by 2025 [65].

Hypertension is a multifactorial disease, and the pathogenesis results from the interaction have genetic, epigenetic, and environmental factors: among these, age is the most critical risk factor. Epidemiological studies revealed that the prevalence of hypertension increases from 27% in patients younger than sixty years old to 74% in those older than eighty years old [66]. Ground-breaking discoveries on the molecular mechanisms of ageing have unveiled several mechanisms involved in age-related hypertension: endothelial dysfunction has a key mechanistic role in initiating and maintaining arterial hypertension.

The endothelial function is mainly regulated by molecular mediators, such as NO and prostaglandins, and mechanical stimuli, such as fluid shear stress (FSS). Although they have been less investigated, mechanical stimuli have a fundamental role in modulating the delicate equilibrium between endothelial function and dysfunction. FSS varies greatly based on the magnitude, direction, and flow speed. Accordingly, FSS can be distinguished in steady laminar, disturbed laminar, oscillatory, and turbulent [67]. Endothelial cells are critical sensors of FSS, and they undergo morpho-functional modifications as a physiological response. However, turbulent FSS can stimulate pathological endothelial remodeling, leading to an increased risk of developing cardiovascular diseases, such as atherosclerotic plaque formation and hypertension [68].

Aging is associated with significant structural and mechanical alterations of vascular endothelium, including the increase of intimal-to-media thickness, aortic length and circumference, and stiffness as well as the reduction of elasticity and distensibility and endothelial capacity to respond to mechanical stresses. All these mechanisms contribute to age-related hypertension. Chala et al., in an in vitro model of endothelial senescence, showed that endothelial cells have a reduced capacity to adapt to the local hemodynamic conditions [69] functionally. Interestingly, recent experimental studies showed that disturbed flow induces endothelial senescence. Thus, vascular FSS could be a critical determinant in regulating vascular senescence [70].

#### Causative Mechanisms of Endothelial Alterations in Hypertension

Several molecular pathways underpinning the structural and functional endothelial alterations have been described in age-related hypertension, including Sirtuins, oxidative stress, and the renin-angiotensin-aldosterone system (RAAS).

Sirtuins are a family of proteins with mono-ADP-ribosyltransferase and deacetylase activity, initially described as class III histone deacetylases [71]. It consists of seven members with different cellular localization and functions. Sirtuins have a protective effect against the decline in cell function. Specifically, SIRT1 is highly expressed in the endothelial cells of arteries, veins, and capillaries, and plays a critical role in preventing endothelial dysfunction [72]. It has been shown that the SIRT1 gene is downregulated during aging. In vivo and in vitro studies showed that the overexpression of SIRT1 slows down endothelial senescence [73].

As stated above, oxidative stress is a hallmark of aging and plays a prominent role in orchestrating age-related endothelial dysfunction. The primary source of ROS is mitochondrial oxidases, NADPH oxidases (NOX), and eNOS. Data from in vivo and in vitro indicate a leading role of NOX [74,75]. NOX is a family of transmembrane enzymes consisting of seven members. Among these, Nox1, Nox2, Nox4, and Nox5 are mainly expressed in the vascular system. The increase in NOX activity has been described in the experimental model of ageing [76]. Noteworthy, the age-dependent increase of NOX is accompanied by the alteration of several molecular pathways, including the improved activation of RAAS, which represents a key regulator of blood pressure. Despite their undiscussed importance in physiological and pathological vascular function, a few studies explored mechanistic insight into the role of NOX in aging. Age-related oxidative stress results from the imbalance between ROS production and degradation. Indeed, aging promotes ROS production while inhibiting antioxidant systems with the net increase of ROS levels. ROS stimulates cellular pathways, leading to aberrant cell signaling and post-translation modifications, such as phosphorylation and oxidation, which impair fundamental cellular processes [77]. The reversible post-translational modifications are critical for regulating protein function (activation or inactivation). Specifically, ROS activate molecular pathways, leading to vascular remodeling, inflammation, and hypertension.

Additionally, ROS can block the Sirt1 function and reduce NO concentration by increased quenching and eNOS inhibition. Overall, all these mechanisms contribute to endothelial dysfunction and related hypertension.

A mutualistic relationship between oxidative stress and RAAS has been described [78]. RAAS is the critical regulator of blood pressure and consists of three main components: renin, angiotensin II, and aldosterone [79]. Furthermore, in vitro studies showed that angiotensin II stimulates the production of superoxide anions, while the inhibition of RAAS is associated with a reduction of oxidative stress [80]. Thus, robust evidence supports a strict relationship between RAAS and oxidative stress, underpinning age-related hypertension.

In the last decade, a role for endothelial progenitor cells (EPC) has emerged [81]. EPCs are a heterogeneous cellular population originating and residing in different organs and tissues, including vascular endothelium. After an injury, they are involved in vascular remodeling by replacing the dysfunctional endothelial cells. It has been shown that aging and hypertension are independently associated with reducing EPCs [82]. Furthermore, cumulating literature evidence shows that oxidative stress induces the reduction of EPCs number. Thus, the interplay among oxidative stress reduced NO bioavailability and decreased EPCs number creates a vicious circle, boosting endothelial dysfunction.

Beyond the well-known role of hypertension in the increased risk of stroke, coronary heart disease, sudden death, heart failure, and peripheral artery disease, age-related hypertension predisposes the onset of cognitive and functional decline, resulting in dementia and physical frailty, respectively. It should be noted that hypertension is commonly underdiagnosed in patients older than 80 years. Indeed, the evaluation of such clinical conditions in the elderly is hampered by several factors, including the poor compliance of the patient and the presence of multiple comorbidities which could hide hypertension [83]. Thus, there is an urgent need to appropriately manage hypertension in the elderly, from diagnosis to treatment.

### 3.2. Endothelial Dysfunction in Diabetes

Type 2 diabetes mellitus (T2D) is a metabolic disease characterized by reduced insulin sensitivity by target organs or reduced insulin production by pancreatic beta cells. Whatever the initial pathophysiologic features, the reduction in insulin sensitivity leads over time to an increase in insulin production to cope, and subsequently to depletion and reduction in pancreatic beta-cell stock due to exhaustion, resulting in hyperglycemia. Certainly, given the pleiotropic effects of insulin, because of the altered glucose metabolism and the effects on lipid metabolism (insulin is an anabolic hormone), T2D is often associated with obesity. However, this association is not always present: there is the nondiabetic obese patient, and there are diabetic subjects who remain at an average weight throughout their life [84].

Undoubtedly, aging promotes the onset of metabolic disorders, and this is the fundamental reason why both T2D and obesity are more frequent in the aging population and why the two diseases often occur together: the senescence that affects other cells in the body does not spare pancreatic beta cells, which become less efficient and more likely to fail as the time passes.

In addition to this, several studies have documented the presence of senescent beta cells in the pancreatic islets of donors with T2D and subjects with high BMI, further demonstrating that several factors, including dysmetabolism, play a crucial role in aging [85].

Hyperglycemia constitutes a harmful condition for the homeostasis of cells, including endothelial cells, and consequently represents the characteristic trigger of diabetes on vascular pathologies. In addition, high serum glucose levels cause functional and metabolic changes through reduced nitric oxide bioavailability, increased oxidative stress, and induction and acceleration of cellular senescence through direct action on the NF-κB pathway.

Under hyperglycemic conditions, the induction of transcription of the gene for the NF-κB factor appears to depend on the activation of molecules of the guanosine triphosphate (GTP)-ases family, which in turn promotes the induction of transcription of inflammatory mediator genes such as IL-1 β, TNF-α, protein kinase C, and adhesion molecules of the cellular adhesion molecule (CAM) family, which facilitate the binding of circulating cells (namely monocytes and T lymphocytes) to endothelial cells.

Atherosclerosis in the diabetic subject is therefore strongly catalyzed by hyperglycemia: on the one hand, inflammation worsens plaque formation as it encourages the oxidation of low-density lipoproteins, and on the other, the reduction in nitric oxide bioavailability, due to sustained endothelial damage, causes hemodynamic alterations and increased shear stress, which are the foundations of vascular pathologies.

In healthy subjects, vasoregulation results from a delicate balance between the vasoactive substances produced by the endothelium and the shear forces exerted by the blood flow. Thanks to continuous feedback, the mechanotransduction of these shear forces allows the endothelium to produce vasodilating substances such as nitric oxide or prostacyclin (PGI2), or vasoconstrictive substances such as endothelin (ET) as required. Sulistyowati et al. documented that hyperglycemia impairs this mechanism, as it disrupts the functioning of endothelial cells by reducing endothelial nitric oxide synthase (eNOS) activity and disrupting mechanotransduction activity, generating the hemodynamic abnormalities discussed above [86].

Concerning nitric oxide, its production in endothelial cells depends on the phosphorylation of the enzyme eNOS by serine/threonine kinases acting coordinately in the insulin signaling pathway; hyperglycemia alters the binding and activation of the insulin receptor, thus leading to an impairment of this molecular mechanism and a reduction of nitroxide [87].

Hyperglycemia impairs endothelial function through a variety of mechanisms: through induction of advanced glycation end products (AGEs) formation, increased expression of receptors for AGEs (RAGE), increased polyol production, and hyperactivation of the hexosamine pathway [88].

The genesis of AGEs causes structural and functional alterations and results in receptor recognition abnormalities of matrix components. In addition, the interaction between ligand (AGE) and its receptor (RAGE) results in increased superoxide anion production that promotes macrophage-mediated inflammatory vascular damage [89,90].

**Figure 1:**
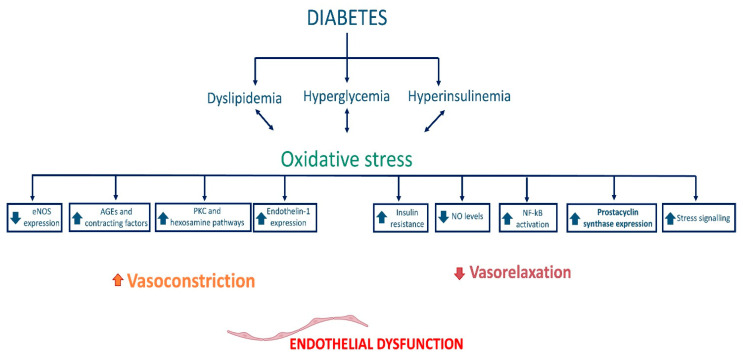
Pathophysiological elements of vascular damage in T2D, with a focus on the dysregulation of the balance between factors regulating vasoconstriction and vasodilation. Endothelial nitric oxide synthase (eNOS), advanced glycation end products (AGEs), protein kinase C (PKC), nitric oxide (NO), and nuclear factor kappa B (NF-κB).

Moreover, the increase in AGEs in the bloodstream reduces nitric oxide synthesis by endothelial cells by reducing the expression of the enzyme eNOS and, consequently, the bioavailability of this vasodilation mediator, conversely facilitating the synthesis of endothelin-1, which mediates vasoconstriction (Figure 1) [91,92].

Regarding polyols and the hexosamine pathway, it should be remembered that hyperglycemia alters glucose metabolism by glycolysis. Therefore, an excess of this metabolite causes the activation of alternative metabolic pathways, such as those mentioned above [93].

In addition, the oxidative stress caused by hyperglycemia induces DNA damage. It promotes the production of ADP-ribose polymer through the activation of nuclear polymerase (PARP), resulting in reduced activity of glyceraldehyde-3-phosphate dehydrogenase, a key enzyme in glucose metabolism, through the cells’ preferred metabolic pathway. This increases the levels of all upstream glycolytic intermediates that trigger the damage mechanisms mentioned above. Oxidative stress, therefore, results from many metabolic alterations that, although established as a result of different preconditions, end up triggering the same abnormalities and cellular damage [88].

The glucose excess and glucotoxicity expressed so far represent part of the determinants of vascular damage, as diabetes mellitus is a dysmetabolic disease “par excellence” and leads to alterations in lipid metabolism: the excess of free fatty acids (FFAs) due to excessive release from adipose tissue and reduced utilization by skeletal striated muscle induces lipotoxicity by molecular mechanisms similar to those already discussed. FFAs induce ROS production by vascular tissue by increasing the expression of the enzyme NADPH oxidase and mitochondrial uncoupling [94].

The increase in the bioavailability of superoxide anion leads to the inactivation of important enzymes with antiatherogenic action, such as eNOS, favoring persistent vasoconstriction; moreover, the consequence of the increase in ROS is a reduction in intracellular glutathione, which constitutes a protective factor of the cell against oxidative damage, so that its reduction is both cause and consequence of oxidative stress itself. Undoubtedly, lipotoxicity has, as a consequence, the activation of the inflammatory cascade and the increase in NF-κB gene transcription, resulting in an increase in inflammation mediators that impacts the vascular district [95].

All the conditions described so far constitute the cornerstone for understanding the relevance of diabetes mellitus as a vascular pathology: metabolic alterations, oxidative stress, and the increased inflammatory response are the pivot and the tip of the iceberg in the pathophysiological cascade of vascular pathologies in the elderly, but the trigger of damage and its perpetuation as an expression of dysmetabolism is the submerged part of the enormous glacier, the element on which to reflect in order to introduce therapies aimed at correcting individual pathways to have a formidable impact on cardiovascular pathology downstream.

### 3.3. Endothelial Dysfunction in Cerebrovascular Diseases

Endothelial dysfunction is considered to be one of the most relevant pathogenetic factors for vascular diseases, and it certainly is for cerebrovascular diseases. Indeed, atherosclerotic vascular diseases of the brain district are a paradigmatic example of this.

The mechanisms that cause the development of the cerebrovascular disease have similar characteristics to those in other districts: oxidative stress due to increased production of reactive oxygen species, low-density lipoprotein (LDL) oxidation and the evolution of incipient atherosclerotic lesion, chronic inflammation, and alterations in vascular tone play a crucial role. Nevertheless, a morbid condition involving and damaging small cerebral vessels is of recent interest. This condition recognizes endothelial dysfunction as one of its most significant causative factors: age-related cerebral small vessel disease (ArCSVD). Cellular senescence and chronic inflammation are crucial elements in the ageing of the organism and are certainly so in cerebral damage; the deterioration of the central nervous system is continuous and progressive in the elderly, and the decline of the cerebral vascular circulation constitutes an essential element of this disease.

Several factors contribute to the genesis of brain damage in the elderly subject, among them immunosenescence and chronic inflammation. However, at the cellular level, the dysfunctional endothelial cells determine the beginning of those abnormalities that will catalyze neurodegeneration.

Senescent endothelium plays a part in regulating vascular tone, remodeling, and the consequent abnormal activation of the coagulation cascade that characterizes ischemic brain damage [96].

The loss of vascular flow regulation occurs as a consequence of inflammatory damage and excessive oxidative stress, which are critical actors of the ageing organism. Furthermore, there is a reduction in the bioavailability of nitric oxide because various factors (such as arterial hypertension and increased levels of angiotensin II) induce transcriptional and post-transcriptional modifications of the eNOS enzyme by reducing its biological activity; all of this favors an imbalance towards permanent vasoconstriction and thus induces a loss of self-regulation of the cerebral circulation, which is fundamental for brain homeostasis [97].

Additionally, in the brain district, chronic inflammation causes the overexpression of inflammatory mediators such as IL-1 β, IL-6, and TNF-α due to the increased expression of the NF-κB gene, leading to the promotion of oxidative stress and the resulting cellular damage: the accentuated oxidative stress further reduces the expression of the eNOS enzyme and the bioavailability of NO, triggering a vicious circle that worsens and amplifies the cerebral hemodynamic alterations [98].

The macroscopic consequence of what has been said so far is the rarefaction of the cerebral vascular tree. Therefore, the reduction in the perfusion of some regions of the cerebral parenchyma is responsible for the increase in perivascular spaces and the genesis of lacunar infarcts that lead to those ischemic abnormalities that result from vascular remodeling.

In the context of cerebral vascular pathologies, endothelial dysfunction plays another fundamental role: the endotheliocyte is a constituent of the blood–brain barrier (BBB), and the structural and functional damage to this cell results in irreversible damage to the BBB and thus to cerebral homeostasis [99].

Indeed, the pathogenesis of BBB disruption is complex. However, some studies have documented that chronic exposure of the endotheliocyte to increased shear stress, in subjects with hypertension, can result in a loosening of tight junctions and thus an increase in barrier permeability by cells of the bloodstream, especially cells of the immune system (which are also senescent) that can infiltrate the brain and trigger and prolong that state of chronic inflammation that can promote vascular remodeling by the mechanisms described above, since increased permeability allows the passage of antigens and harmful stimuli that should not typically reach the brain district [100,101].

## 4. The Impact of Chronic Inflammation and Endothelial Changes on Age-Related Diseases

The pivotal element behind cellular ageing is oxidative stress. In the mid-1950s, the “free radical theory” was identified and took hold [102]. The reactive oxygen species (ROS) produced by the enzyme NADPH (reduced nicotinamide adenine dinucleotide phosphate)-oxidase and by the mitochondria lead to alterations in the molecular processes of senescent cells, stimulating the dysfunction of endothelial cells and feeding the chronic inflammation as discussed above, leading to irreversible changes in the vascular system [103,104].

**Figure 2:**
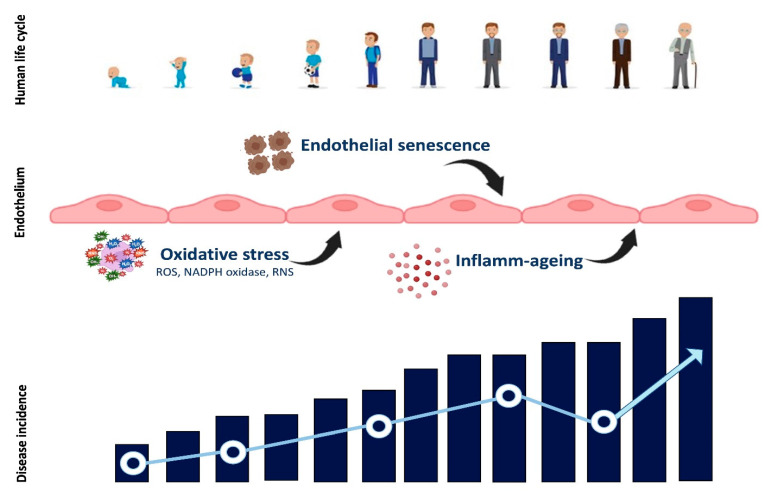
The evolution of vascular damage in relation to ever-increasing endothelial dysfunction as man ages, showing the pathogenetic factors that favor cellular damage.

This premise is necessary because cardiovascular and cerebrovascular diseases have an enormous epidemiological impact and colossal health costs. Moreover, in the coming years, they will constitute an even more significant problem considering the ageing of the world’s population (Figure 2).

As already extensively described above, the cornerstones of ageing are chronic inflammation and cellular senescence.

In addition to the molecular mechanisms already known, scientific research is increasingly looking for elements that can correlate the changes undergone by individual cells and the effects on the entire organism in terms of senescence.

Recent evidence has documented that, in addition to self-damage associated molecular patterns (DAMPs) and non-self pathogen-associated molecular patterns that trigger inflammation via Toll-like and NOD-like receptors [16], there are receptors that regulate intracellular signaling, such as the Notch and Klotho/FGF23 pathways, which play a part in the onset and maintenance of inflamm-ageing. However, some biochemical aspects remain to be elucidated [105,106].

Even if these pathways play a part in the survival of the organism against harmful stimuli during youth, with ageing, their chronic proinflammatory activity (expressed through the increased production of cytokines including IL-1 and IL-18) is the driving force behind the involution and decay to which organs and tissues are subjected [16].

The modulation of each individual’s inflammatory activity is not only regulated at the genetic level: the production of cytokines and other inflammatory mediators is also regulated at the epigenetic level so that the modulation of RNA transcription and translation also occurs through DNA methylation, histone modifications, and regulation of long non-coding RNAs; this suggests that, as for many diseases, and also for ageing, a complex synergetic relationship between genetics and environment exists [107].

### 4.1. Metaflammation

Concerning the factors involved in the modulation of the inflammatory response, researchers have recently found that, in the genesis and development of age-related diseases, in addition to the known factors, there is an excess of nutrients and calories that determine the phenomenon called ‘metaflammation’. It seems that the mechanisms underlying ‘metaflammation’ feed the mild chronic inflammation that characterizes ageing (inflamm-ageing), a kind of dog biting its tail: excess calories increase the extent of inflammation, which in turn accelerates ageing and the decay of the molecular mechanisms that regulate metabolic processes, favoring the onset of dysmetabolic diseases in the elderly [108,109]. In contrast, a reduction in caloric intake leads to successful aging and maintained endothelial function [30]. This is probably due to the fact that an intake of food protects against macromolecular damage due to redirection of glycolysis [31,110].

The link between these processes is the gut microbiota: the bacteria that populate the human gut modify nutrients into metabolites that reach all cells and influence their bioenergetic activities. With age, this population of bacteria undergoes profound changes, and the metabolites produced by the processing of ingested food change. This forms the basis of nutrition-related inflammation, so that the transfer of bacteria from the gut of old animals into the gut of young ones results in increased intestinal permeability, dysfunction of macrophages, increased production of inflammatory cytokines such as TNF-alpha, and alteration of the adaptive immune response [111,112].

In further support of the above, scientific literature suggests that in humans, the increased presence of Proteobacteria (characteristic of ageing) is associated with increased serum concentrations of inflammatory mediators such as IL-6 and IL-18 [113].

Moreover, aging assumes that the bacterial population in the gut becomes more abundant and less diversified, a characteristic that probably plays a detrimental role for the organism; on the other hand, in successfully aged individuals, the microbial flora is varied, suggesting that the diversity of bacterial populations is one of the factors that contribute to promoting longevity and counteracting the onset of age-related dysmetabolic diseases [114,115].

All this has an important practical implication: inflamm-ageing promotes atherosclerosis independently, but it interacts with classic cardiovascular risk factors (hypertension, obesity, type 2 diabetes mellitus) that represent a significant inflammatory stimulus, causing the onset of cardio- and cerebrovascular disease by an endless vicious cycle. These plans overlap that scientists debate whether inflamm-ageing exists in the absence of dysmetabolic diseases (diabetes or obesity) [116].

In the obese population, excess nutrient intake promotes an inflammatory microenvironment: after a meal, triglyceride-rich lipoproteins promote the expression of adhesion molecules, cytokines, and pro-oxidant agents by leukocytes and endothelial cells that induce vascular damage [117]. Furthermore, cell hypertrophy occurs at the adipose tissue level in these individuals, resulting in local hypoxia that can alter the protective pathways of AMP-activated protein kinase (AMPK) and sirtuin proteins (SIRTs), leading to an imbalance toward proinflammatory factors and constituting a catalyst for cardiovascular risk. Conversely, caloric restriction activates mechanisms to protect against inflammatory damage that prevent the onset of these diseases [118,119]. In addition, the proinflammatory environment promotes insulin resistance, a well-recognized cardiovascular risk factor in the elderly; this explains why the cardiovascular risk of the elderly with diabetes mellitus is higher than that of a young person [120,121].

This consideration provides the background for further investigation of the relationship between aging, inflammation, insulin resistance, and cardiovascular risk: elevated serum levels of cytokines and mediators of inflammation, a product of inflamm-ageing and metaflammation, result in significant recruitment of immune cells to insulin-responsive tissues such as fat and muscle, inducing oxidative stress that reduces the expression of insulin receptors [122,123].

This results in increased concentrations of glucose and free fatty acids that over time produce the switch from metabolically sustainable obesity to metabolic syndrome and all the consequences it involves [124].

The excess of these products in the blood circulation induces endothelial dysfunction and smooth muscle cell dysfunction of the vessel wall, promoting the development of hypertension: macrophages, the key players in this process, as a consequence of the proinflammatory set-up established, produce abundant reactive oxygen species (ROS) that inhibit endothelial nitric oxide synthase (eNOS) activity and reduce the bioavailability of nitric oxide (NO), stimulate extracellular matrix metalloproteases, and promote the expression of adhesion molecules that allow the recruitment of other immune cells, resulting in vasoconstriction and vascular remodeling [125].

The increase in mediators of inflammation (TNF-α, IL-1 β, caspase-1) and the mirror reduction in anti-inflammatory cytokines (IL-10 and adiponectin) accelerates atherosclerotic plaque formation, accentuates prothrombotic risk, and supports increased cardiovascular risk by stimulating extracellular matrix remodeling and promoting arterial stiffness [126].

Inflamm-ageing and classic cardiovascular risk factors (diabetes, hypertension, obesity), therefore, are unique but interconnected elements, endowed with their specific weight in the genesis of the elderly’s diseases but synergistic when present together.

Aging, as a whole, is a complex phenomenon: its epicenter is the loss of each cell’s ability to respond to external stresses while maintaining homeostasis. Speaking of vascular damage in the elderly, the chain of events leading to the alterations already analyzed begins with oxidative stress and the damage produced by ROS. However, how is it that aging results in this decline? What changes, compared to the young subject, in vascular homeostasis?

To answer this long-standing question, one of the most studied models is nuclear factor erythroid 2-related factor 2 (Nrf2). It is a redox-sensitive transcription factor implicated in antioxidant response and repair of molecular damage generated by ROS [127].

Aging is associated with a reduced Nrf2 expression and a weak anti-inflammatory response, resulting in cellular damage promoted by oxidative stress and vascular alterations [128]. Caloric restriction, in contrast, appears to implement Nrf2 expression and the body’s ability to respond to oxidative stimuli, preventing the onset of those diseases that identify oxidative stress as the primary motive [129]. This again confirms the interplay between caloric excess and inflammation in the setting of the elderly organism and demonstrates how the diseases of the elderly have an inflammatory basis that is not only related to aging but also dysmetabolic changes, the fuel of chronic inflammation.

### 4.2. Loss of Proteostasis

Beyond the mechanisms described so far, scientists are interested in better characterizing the aberrations in the aging organism. Among these, one of the most suggestive is the loss of proteostasis.

Indeed, evidence suggests that proteasome stability and the integrity of the proteostasis network are essential for successful cardiac aging [130,131].

Disruption of the balance between protein synthesis and degradation appears to be a critical element in the onset of vascular disease: an increase in misfolded protein aggregates has been documented to be associated with the onset of cardiovascular disease [132].

Ageing significantly impairs the key players in maintaining cardiovascular proteostasis, specifically the ubiquitin-proteasome system, chaperones, and the lysosome-autophagy system. Chaperones play a crucial role in the transport, assembly, and degradation of other proteins, and help prevent misfolding and pathological aggregation. In the elderly, molecules produced as a result of vascular damage downregulate the expression of HSP-70 (70 kilodalton heat shock proteins) in the vascular system. In addition, mitochondrial damage and reduced intracellular ATP levels hamper the activity of ATP-dependent chaperones [133].

Autophagy mechanisms (micro- and macroautophagy and chaperone-mediated autophagy), under physiological conditions, promote the degradation of harmful molecules and the recycling of degraded components to ensure the energy efficiency of cells. Several pieces of evidence suggest that deficiencies in the mechanisms regulating autophagy are responsible for age-related vascular damage, not least because, in experimental systems where autophagy-stimulating substances (such as spermidine or trehalose) have been used, a reversal of arterial envelopment has been observed. However, in clinical reality, this has not resulted in effective pharmacological interventions [134,135].

Of course, the ubiquitin-proteasome system also has a pivotal function, as it contributes to the degradation of proteins that are no longer useful to the cell or are damaged through proteolysis. As already mentioned, the activity of this proteolytic system decreases with age, and in experimental animal studies, a reduction in its activity was observed in the aged rat heart [136].

The pathway that leads to the onset of cardiovascular disease in the elderly is very jagged and complex, and as described so far, presupposes the co-presence and synergistic activity of factors that push in the direction of malfunctioning various tissues and organs. Chronic inflammation, endothelial dysfunction, reduced bioavailability of nitric oxide and the consequent increased oxidative stress, alterations in the gut microbiota and the caloric excess typical of obese older adults with comorbidities, mitochondrial dysfunction and altered proteostasis: each of these factors is linked by an indissoluble thread to the others and constitutes a piece of the jigsaw puzzle of old age. Many mechanisms remain to be discovered and characterized. However, their understanding and the synoptic view of old age will increasingly make it possible to develop new drugs and new therapeutic strategies to counteract these processes by impacting pathologies and cardiovascular risk, making it possible to slow down, as far as possible, the endless passing of time and the deterioration it entails.

## 5. Current Evidence and Future Perspectives: Can This Progressive Decline Be Prevented?

Inflamm-ageing constitutes the cornerstone of age-related diseases. On this basis, scholars sought to understand whether inhibition of the underlying mechanisms could interfere with the development of diseases in the elderly and, more specifically, cardiovascular diseases.

Before and more than inflamm-ageing, inflammation, in general, constitutes the fertile ground for the onset of vascular damage: several studies have shown that individuals with chronic extra-cardiac inflammation, e.g., suffering from chronic inflammatory diseases such as rheumatoid arthritis or psoriatic arthritis, have a higher risk of developing cardiovascular disease (adjusted relative risk of two for myocardial infarction) and thus a higher mortality rate than those who are not affected, and this is due to the contribution of systemic inflammation as an independent risk factor for cardiac and vascular diseases [137,138].

The results of several scientific studies support this: numerous prospective cohort studies have documented that high serum levels of high-sensitivity C-reactive protein (hsCRP) and other inflammation markers are independent risk factors for cardiovascular disease [139].

The therapeutic approaches from which all this began to be understood were carried out through the use of statins: the JUPITER trial involving the use of rosuvastatin in primary prevention in patients who had hsCRP levels above the population median and LDL (low-density lipoprotein) levels below 130 mg/dL, clearly not suffering from systemic inflammatory diseases, documented that there was a reduction in cardiovascular events compared to controls and that there was a reduction in LDL and hsCRP levels [140].

Subsequently, the PROVE-IT trial also documented a reduction in cardiovascular events by reducing LDL and inflammatory burden; however, like the previous study, it was unclear whether the reduction in cardiovascular accidents was due to action on LDL or action on inflammatory mechanisms. These data raised the suspicion that inflammation played a role, without clarifying whether it was possible to reduce the incidence of cardiovascular disease by acting solely on the extent of inflammation [141].

In this regard, subsequent trials have considerably impacted the scientific literature by which drugs capable of counteracting inflammation in a more specific way have been studied, thus allowing the direct effect in terms of cardiovascular risk to be assessed.

The CANTOS study (Canakinumab anti-inflammatory thrombosis outcomes study) evaluated the administration of canakinumab, an inhibitor of a potent inflammatory mediator such as IL-1 beta, in a cohort of 10,061 patients with previous myocardial infarction who had serum hsCRP levels of 2 mg/L or more and were already receiving optimized medical therapy, including statin treatment: subcutaneous administration of the drug every three months reduced serum hsCRP levels in treated subjects compared with controls without interfering with lipoprotein levels. At a median follow-up of 3.7 years, statistical significance was achieved for the primary endpoint (reduction in nonfatal myocardial infarction, nonfatal stroke, and cardiovascular mortality) and the secondary endpoint (unstable angina with the urgent need for revascularization). In addition, all-cause mortality was essentially superimposable between the two groups; fatal infections were more common in canakinumab-treated subjects than in the placebo group [142].

Another significant trial, for reasons opposite to the previous ones, was the CIRT (Cardiovascular Inflammation Reduction trial), a randomized placebo-controlled trial that evaluated the administration of methotrexate in patients with previous myocardial infarction or multivessel coronary artery disease who were diabetic or had metabolic syndrome; the trial was stopped after two years due to futility, as no reduction in the serum concentration of key inflammation mediators or reduction in cardiovascular risk was observed [143]. This gives us food for thought that, to hinder the mechanisms of inflammation, it is necessary to selectively act on specific targets and consequently observe significant effects on cardiovascular risk.

Among the drugs that have received attention over the years, we must mention colchicine: it acts by inhibiting tubulin polymerization, interferes with the assembly of the multimeric NLRP3 inflammasome, and hinders the expression of chemokines that play a role in the genesis of inflammation. In the LoDoCo trial, 532 patients with stable coronary artery disease were randomized and divided into two groups, with one group taking colchicine in addition to previous medical therapy and the other group not for a time of at least two years. Patients receiving colchicine had fewer cardiovascular events over time without paying the price of significant side effects; however, the study did not receive enormous prominence in light of the small sample size [144].

The most recent COLCOT trial (COLchicine Cardiovascular Outcomes) evaluated the effects of colchicine on cardiovascular events and drug tolerability in a population of 4745 patients after nonfatal myocardial infarction recruited within 30 days of the event and at 22.6 months after the event, there was a reduction in resuscitated cardiac arrest, myocardial infarction, stroke, and other endpoints compared with the placebo group, while there was no difference for all-cause mortality [145].

The crux of these studies (Table 1), from which our thinking moves, is that inhibition of inflammatory mechanisms certainly contributes to reducing cardiovascular risk if it is true, as we have said that the inflammatory burden plays a key role in the genesis of vascular disease. Nevertheless, inhibition of inflammation implicates an increased risk of incurring serious infections, inflammation being the body’s natural response to infectious stimuli, and likely an increased risk of neoplastic diseases.

The prospect before us is affluent in suggestions and insights regarding new therapeutic strategies, even considering recent acquisitions in the field of research.

Recently, an element that links aging, inflammation, and cardiovascular risk has been identified and is a singular novelty: clonal hematopoiesis. With age, hematopoietic stem cells in the bone marrow acquire somatic mutations that result in the accumulation, in the peripheral blood, of mutant leukocyte clones. In those over the age of 70, there is a chance of finding these clones, amounting to at least 2% of circulating leukocytes [146].

The mutations occur on about 40 well-characterized genes implicated in leukemic transformation. However, only a tiny percentage of these individuals go on to acute leukemia because three or more mutations are needed in the same clone for its development. All those who do not develop leukemia, therefore, will have a proportion of clones that do not have enough mutations to go on to leukemic transformation and constitute that condition called “clonal hematopoiesis of indeterminate potential” (CHIP) [147].

Interestingly, while individuals with CHIP have a 0.5–1% annual chance of developing acute leukemia, they likewise have a 40% increase in cardiovascular risk regardless of traditional risk factors [148]. All this has been supported by animal studies, especially on rats with induced mutations on some of the CHIP-promoting genes, in which accelerated atherosclerosis and a higher occurrence of vascular disease was observed [149].

Thus, it appears that the presence of CHIP does not simply accompany aging but plays a crucial role in the onset of its diseases. Moreover, scientific research has enabled even better characterization of the CHIP condition. For example, in animals where the genesis of these mutant clones has been induced, overexpression of proinflammatory genes (such as the gene for the NLRP3 inflammasome) and increased onset of myocardial infarction, stroke, and heart failure and, over time, death from heart failure have been observed [150].

The prominence of inflammatory mediators, and the centrality of the inflammasome, have led scholars to set up studies to evaluate the possibility of inhibiting certain checkpoints from fighting the diseases of aging, so much so that there are ongoing trials to evaluate the efficacy of molecules that can inhibit the inflammasome from reducing cardiovascular risk. Blocking the inflammasome and reducing the blood concentrations of its products, first and foremost IL-1 beta and IL-18, could be an exciting target of future therapies, perhaps using monoclonal antibodies to tailor actions to specific targets, and following the trail blazed by the CANTOS study that was a significant contribution to modern science. Indeed, it will need to be verified that inhibition at different levels of the inflammatory cascade does not have spin-offs in terms of the development of neoplastic diseases or complications of systemic infections such as sepsis or that the risk–benefit ratio associated with taking these drugs in primary prevention is favorable. However, compared with the past, today there are suggestive and fascinating pathways that hint at the possibility of counteracting the passage of time and the onset of age-related diseases [151,152,153,154,155,156]; after all, man has always sought to counteract the decline of his body through the fabled search for the elixir of long life. More pragmatically, the development of drugs of this type may enable successful aging.

## Figures and Tables

**Table 1:** Summary of trials that have evaluated the use of anti-inflammatory drugs with a summary of the results of each, a prerequisite for the future of scientific research.

TRIALS	YEAR	SIZE	DRUG	POSOLOGY	CONCLUSIONS
**PROVE-IT**	2004	1018	ATORVASTATIN, PRAVASTATIN	80 mg in the former, 40 mg in the latter	Atorvastatin 80 mg was superior to pravastatin 40 mg regarding the dual objectives of aggressive LDL-C and CRP reduction, targets capable of leading to a reduction in cardiovascular events;
**JUPITER**	2008	17,603	ROSUVASTATIN	20 mg	the statin-induced reduction in cardiovascular events was due to a reduction in vascular inflammation (measured by a reduction in hsCRP) but also to a reduction in LDL levels;
**CANTOS**	2017	10,061	CANAKINUMAB	150 mg every three months	reduction of cardiovascular events by lowering hsCRP levels (by blocking IL-1 β targeting), independent of LDL levels;
**CIRT**	2019	4786	METHOTREXATE	15–20 mg weekly	premature blockade for ineffectiveness in reducing inflammatory mediators and cardiovascular events in patients with stable atherosclerosis;
**COLCOT **	2019	4745	COLCHICINE	0.5 mg once daily	reduction in the recurrence of cardio- and cerebrovascular events within 30 days of the primary event;
**LoDoCo**	2020	532	COLCHICINE	0.5 mg once daily	reduction of cardiovascular events in patients with chronic coronary artery disease.
